# Extracellular Vesicles in Smoking-Mediated HIV Pathogenesis and their Potential Role in Biomarker Discovery and Therapeutic Interventions

**DOI:** 10.3390/cells9040864

**Published:** 2020-04-02

**Authors:** Sanjana Haque, Sunitha Kodidela, Kelli Gerth, Elham Hatami, Neha Verma, Santosh Kumar

**Affiliations:** College of Pharmacy, University of Tennessee Health Science Center, 881 Madison Ave, Memphis, TN 38163, USA; shaque8@uthsc.edu (S.H.); skodidel@uthsc.edu (S.K.); kgerth1@uthsc.edu (K.G.); ehatami@uthsc.edu (E.H.); neha.verma@smail.astate.edu (N.V.)

**Keywords:** extracellular vesicles, cigarette smoking, HIV, HIV-associated neurocognitive disorders

## Abstract

In the last two decades, the mortality rate in people living with HIV/AIDS (PLWHA) has decreased significantly, resulting in an almost normal longevity in this population. However, a large portion of this population still endures a poor quality of life, mostly due to an increased inclination for substance abuse, including tobacco smoking. The prevalence of smoking in PLWHA is consistently higher than in HIV negative persons. A predisposition to cigarette smoking in the setting of HIV potentially leads to exacerbated HIV replication and a higher risk for developing neurocognitive and other CNS disorders. Oxidative stress and inflammation have been identified as mechanistic pathways in smoking-mediated HIV pathogenesis and HIV-associated neuropathogenesis. Extracellular vesicles (EVs), packaged with oxidative stress and inflammatory agents, show promise in understanding the underlying mechanisms of smoking-induced HIV pathogenesis via cell-cell interactions. This review focuses on recent advances in the field of EVs with an emphasis on smoking-mediated HIV pathogenesis and HIV-associated neuropathogenesis. This review also provides an overview of the potential applications of EVs in developing novel therapeutic carriers for the treatment of HIV-infected individuals who smoke, and in the discovery of novel biomarkers that are associated with HIV-smoking interactions in the CNS.

## 1. Introduction

Once a lethal pandemic, HIV has now taken the form of a chronic condition. As of 2018, at least 37.9 million people were living with HIV, with more than a million new cases each year [1]. The majority of these people living with HIV/AIDS (PLWHA) have a life expectancy comparable to healthy adults, which is attributed to remarkable advances in medicine, especially the introduction of combination anti-retroviral therapy (cART) [2,3,4,5]. However, a huge portion of PLWHA have a poor quality of life and suffer from high morbidity and mortality associated with drugs of abuse, including tobacco. More than 40% of PLWHA in the USA are cigarette smokers, which severely affect their life expectancy, reducing the average life span by over 6 years [6,7,8]. Mortality due to non-AIDS related malignancies is almost two-fold higher in HIV-positive smokers, irrespective of the use of cART [9]. Non-adherence to cART and/or attenuated treatment efficacy in HIV-positive smokers could possibly increase the risk of morbidity and mortality [10,11]. Smoking cessation can improve life expectancy, although studies have shown that smoking cessation is hard to achieve [12]. While the exact mechanistic pathway for smoking-mediated exacerbation of HIV pathogenesis is not fully understood, our studies have shown that tobacco smoke aggravates HIV pathogenesis, in part via the induction of cytochrome P450 (CYP)-mediated metabolism and activation of cigarette smoke constituents, resulting in oxidative stress [13,14,15,16,17,18]. In particular, we have demonstrated that benzo(a)pyrene (B(a)p), a potent component of cigarette smoke, exacerbates HIV replication via CYP-induced oxidative stress followed by the NF-κβ pathway [13].

Approximately 50% of PLWHA demonstrate a pattern of cognitive, motor, and behavioral dysfunction, cumulatively termed HIV-associated neurocognitive disorders (HAND) [19,20,21]. In the presence of cigarette smoke, the risk of peripheral neuropathy and HAND in PLWHA increases significantly [7,22,23,24,25]. Some reports demonstrate a conflicting impact of cigarette smoke on PLWHA in terms of neurocognitive disorders [26,27,28], which further strengthens the necessity to study whether cigarette smoking is a causative factor for HAND in PLWHA. 

One possible mechanistic pathway of tobacco smoking-induced HIV pathogenesis and HAND could be the transportation of oxidative stress-related agents and inflammatory modulators via extracellular vesicles (EVs), commonly referred to as exosomes prior to 2018. EVs are biological nanoparticles and are released by almost all cells [29,30]. They are considered as both inter and intra-cellular messengers, able to modify their cargo according to the condition or stimulus affecting the parent cells [31,32], which upon internalization by recipient cells, can modulate the pathophysiological state in those cells [33,34]. EVs play an important role in HIV pathogenesis - either in improving or deteriorating the existing condition; however, the exact role of EVs in HIV pathogenesis is poorly understood [35,36]. Currently, only a handful of studies have investigated the role of EVs in smoking-mediated toxicity in the setting of HIV [37,38,39]. 

The complete eradication of HIV is not currently feasible, with a few exceptions [40,41], due to viral latency in cellular reservoirs, e.g. CD4 T cells, cells of the myeloid lineage (monocytes and macrophages) and dendritic cells [42,43,44,45]. Monocytes and macrophages are considered one of the most suitable cells for studying viral latency due to their long lifespan, as well as their ubiquitous presence throughout the body, including the brain [43,45,46]. EVs derived from monocytes and macrophages potentially have a profound effect on recipient cells [47,48]. For example, we have previously reported that EVs derived from uninfected monocytes protect recipient cells due to the specific packaging of protective elements [39], manuscript under revision). Conversely, HIV-infected macrophage-derived EVs lose this defense capacity, as evidenced by a higher viral load and increased cellular toxicity [39]. Proteomic and cytokine analyses of plasma EVs obtained from HIV-positive and negative smokers demonstrated a differential packaging of proteins in the EVs [37,49]. In addition, macrophage-derived EVs can readily cross the blood brain barrier, suggesting the potential role of EVs in either disseminating or alleviating HIV and HAND pathogenesis [30].

Evidently, there is a strong correlation between cigarette smoking and HIV and/or HAND pathogenesis as demonstrated via EVs. Nevertheless, there are unresolved questions to be answered. For example, what is the true nature/role of EVs in smoking-mediated HIV and HAND pathogenesis? Can we inhibit the viral transfection and oxidative stress through EVs? Is there any therapeutic application of EVs in this context? Can the components of EVs be used as biomarkers for HIV-tobacco smoking interactions that lead to HAND? Very recently, especially in the last five years, more and more studies are being conducted to answer these questions. This niche field has drawn researchers to connect the dots between HIV, cigarette smoking, HAND and EVs, and whether oxidative stress acts as a driving force to exacerbate the conditions. This review will explore possible answers to these questions, emphasizing investigations since 2015 and speculating on the potential role of EVs in HIV pathogenesis and HAND in tobacco smokers, as well as propose therapeutics to combat these conditions.

## 2. Extracellular Vesicles-a Sneak Peek

The discovery of extracellular vesicles (EVs), particularly exosomes-one particular type of EVs, dates back to the 1980s [50,51]. There are various classifications of EVs, including exosomes, extracellular vesicles, microsomes, ectosomes, prostasomes, oncosomes etc. The EVs that are generally formed in the multivesicular bodies within cells are exosomes. These are the nanosized vesicles (≤ 200nm), that capture the functional messages from cells and are thus the primary vesicles of interest [52,53,54,55,56,57,58]. Unfortunately, isolation and purification of exosomes from other sorts of extracellular vesicles released by the cells remain challenging. Thus, most of the isolated vesicles are a mixed population of exosomes, small extracellular vesicles, and microvesicles. Therefore, according to the guidelines provided by the Minimal information for studies of extracellular vesicles (MISEV), it is more appropriate to use the generic term extracellular vesicles (EVs) to designate these vesicles [29]. 

Ultracentrifugation is considered the gold standard method for the isolation of EVs from cell culture media and biological fluid [59]. However, this method has its own limitations that include the requirement for a large volume of starting fluid, co-precipitation of unwanted materials etc. [60]. Several isolation techniques have been developed to overcome these limitations, such as- sucrose gradient centrifugation with iodixanol, size-exclusion chromatography, polymer-based precipitation, antibody-specific EVs separation technique etc. [60,61,62,63,64]. Due to persistent discrepancies in EVs isolation procedures, the MISEV 2014 guideline recommended choosing the isolation method according to the need of the downstream analysis [65]. After successful isolation of EVs, it is equally important to characterize the particles. According to the MISEV 2014 guideline, molecular techniques to identify the presence of at least three or more exosome-enriched proteins (e.g. tetraspanin membrane proteins, heat shock proteins, membrane transporters and lipid-bound proteins), absence of cell-specific proteins (e.g. β-actin, GAPDH) [65]. At least two different biophysical techniques should be performed for biophysical characterization of EVs. These techniques could be optical particle tracking, including Transmission Electron Microscopy and atomic force microscopy [66,67,68]. Comparable data from nanoparticle-tracking analysis, dynamic light scattering, or resistive pulse sensing, are the basic requirements for EVs [65]. The contents of EVs vary greatly depending upon the condition of the parent cell. Thus, apart from characterizing the vesicles, identifying these contents reveals a breadth of information regarding the parent cells. 

The unique nature of EVs is their role as intercellular messengers. The speculation started with the finding that EVs were involved in indirect activation of CD4-T cells by carrying major histocompatibility complex (MHC) and T cell costimulatory molecules [69,70]. In 2007, Valadi et al. took the EV field one-step forward by demonstrating that these vesicles packaged and shuttled mRNA and miRNA to recipient cells for translation to functional proteins [55,70]. Since then, extensive studies have revealed that EVs are released from nearly all kinds of mammalian cells [31,71,72,73,74]. The intricate role of EVs in disease pathogenesis is thus a matter of deep interest. The most studied is perhaps the role of EVs in cancer or tumor pathogenesis [75,76,77,78,79,80,81]. Additionally, EVs have been demonstrated to participate in disseminating pathogens like HIV, HCV, EBV, prions etc. [82,83,84,85,86]. The focus of this review will be in the field of research around EVs in HIV.

### 2.1. Advances Since 2015

#### 2.1.1. Isolation Techniques and Characterization Methods

Recently, the isolation procedure of EVs has seen tremendous development. Rong et al. have demonstrated a sequential ultrafiltration method to isolate two subtypes of EVs (exosomes and microvesicles) from cancer cells. Although the sequential ultracentrifugation method allows for the separation of more purified exosomes, it requires a substantial amount of starting material [87]. Mengxi et al. have developed an integrated acoustics and microfluidics method to isolate EVs from undiluted whole blood, with high purity and yield in an automated system [88]. Furthermore, a commercial column method to isolate EVs is also currently available [89]. However, there is still potential for improvement, in terms of purity, yield, and the cost of the isolation procedure [90]. For the characterization of EVs, MISEV 2018 updated the 2014 guideline and suggested, “Both the source of EVs and the EV preparation must be described quantitatively”, with global quantification. With other guidelines being revised and updated, MISEV stressed that since the research field of EVs is highly diverse and continuously evolving, investigators have the flexibility to divert from the suggested guidelines with appropriate reasoning [29].

#### 2.1.2. EVs as Intercellular Messengers

**Figure 1:**
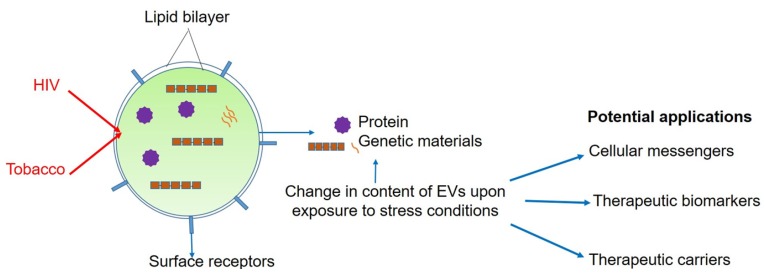
Basic characteristics and potential applications of extracellular vesicles upon exposure to HIV and tobacco smoke.

In addition to isolation techniques, knowledge of the role of EVs in cell-to-cell communication is also evolving rapidly. New insights are coming forward in terms of understanding cellular uptake of EVs. Some EVs demonstrate cell-specific uptake, while others are universally internalized by all cells. The exact reasons for this semi-selective nature is still to be discovered, however, some studies have identified specific receptor binding molecules on the surface of EVs that are responsible for intercellular communication [91]. Depending on the uptake process, the EVs can release their cargos at the plasma membrane of the recipient cells or go through endocytosis to be re-released within cells, with modified characteristics. Whether the EVs will have a functional or phenotypical impact on the recipient cells largely depends on the process by which EVs release their components. Therefore, there is a huge gap in knowledge to fully comprehend the true impact of EV-mediated intercellular communication. However, after internalization and release of cargos, the role of EVs in the physiological modulation of recipient cells is well-established [39,92,93,94,95,96,97]. Interestingly, the physiological effects of EVs are as diverse as their contents. Moreover, the potential of using EVs in biomarker identification has rapidly grown, which expanded from cancer biomarker identification to liquid biopsy in ADME (absorption, distribution, metabolism, and excretion) phenotyping of a drug [98,99,100]. Figure 1 is a diagram depicting basic characteristics of EVs and their potential applications.

## 3. Role of EVs in HIV 

EVs have vital role in HIV pathogenesis. To begin with, EVs and HIV both share a multitude of similarities in terms of size, origination, and cellular release mechanisms [101]. EVs carry viral proteins like Negative Regulatory Factor (nef), transactivator of transcription (Tat) encoding protein, entry receptors like CCR5 etc. [102,103,104]. On the other hand, HIV can utilize the release pathway of EVs to disseminate viral transmission [103]. Again, it is well established that oxidative stress is involved in various stages of the HIV life cycle, including viral replication and the inflammatory response, ultimately leading to the destruction of T cells, as well nuclear and mitochondrial DNA in brain tissue [105,106,107,108,109,110]. However, it is still not clearly understood how these oxidative and immunomodulatory factors are translocated from cells of origin to neighboring cells or even to distant organs. Based on the role of EVs in cell-cell communication, it is hypothesized that EVs carry these agents from parent cells to recipient cells in distant places, including the brain, by crossing the blood brain barrier (BBB). Since 2015, studies have been exploring the potential role of EVs in transporting oxidative stress and immunomodulatory agents in the pathogenesis of HIV and HAND, which will be discussed in detail in the following section.

### Advances Since 2015

It is well documented that HIV-infected populations have elevated levels of EVs in their plasma in comparison to healthy individuals [111]. EVs originated from HIV-infected cells can reveal important information about these cells as well as the phase of the infection. Most importantly, they can provide information about the cell’s exposure to stressors [112,113,114]. Sukrutha Chettimada et al. have shown that the EV cargos provide a wealth of information about immune responses and oxidative stress. Their study not only confirmed the increased level of plasma EVs in the HIV positive patients, but also showed that this increase directly correlated with the increase in oxidative stress marker (cystine, oxidized cys-gly). They also detected other oxidative stress markers such as CAT, PRDX1, PRDX2, and TXN, markers of EVs, immune markers, and Notch4 in plasma EVs by untargeted proteomic analysis [115]. Moreover, they showed reduction in the anti-inflammatory polyunsaturated fatty acid (PUFA) level, which are known to be responsible for sensitizing cells to oxidative stress and eventually leading to cell death and apoptosis [115]. 

The contents within EVs can be used as biomarkers for diagnostic purposes, which would be an excellent non-invasive method [116]. The therapeutic use of EVs as biomarkers has been utilized for cancer therapy for a while now [117]; however, it is still an emerging field in the case of HIV. For instance, EVs from PLWHA are enriched with miR-122 and miR-200a, which are known markers for liver disease [118]. HIV-associated neurocognitive disorder, demonstrated by Aβ deposition, is highly common in PLWHA, which is a well-established biomarker for Alzheimer’s disease. Interestingly, neuron derived EVs from HIV populations show high levels of Aβ, which can serve as a possible biomarker for Alzheimer’s disease in PLWHA [119,120]. Markers of general neurodegeneration, e.g. HMGB1 and NF-L, are also found in these EVs, suggesting their potential role in biomarker identification [120,121]. 

Taken together, EVs shuttle various biomarkers that could prove to be useful in clinical application. Moreover, as EVs can be collected in a noninvasive method without any surgical procedure, this would make them an ideal candidate for diagnostic applications.

## 4. Role of EVs in HIV and Tobacco-Smoking

Cigarette smoke contributes to the deterioration of pre-existing conditions of HIV by various mechanisms, one of them being the CYP-induced oxidative stress-mediated NFκβ pathway [17,18,122,123]. However, other studies challenge these observations, which prompts further investigation [124,125]. It is thought that oxidative stress conditions induced by cigarette smoking trigger the release of EVs, as EVs may play a key role in inflammatory pathways [126] and in some cases may mediate protective signals during oxidative stress, reducing cell death [127]. Our earlier studies revealed a higher expression of CYP1A1 and antioxidant enzyme mRNAs in EVs from CSC-exposed monocytes and point toward a protective role of EVs against CSC-mediated cytotoxicity [39]. Although the exacerbation of HIV pathogenesis via cigarette smoke constituents inducing oxidative stress has been frequently proposed in literatures, the role of EVs in this pathway is scarcely studied until 2015. The next section will discuss about the advances in this field in the last five years.

### Advances Since 2015

Earlier studies have demonstrated that EVs are involved in the shuttling of biomarkers, which could be beneficial as a diagnostic tool. However, it is still largely unknown if a specific biomarker or agent is directly linked to cigarette smoking-mediated HIV pathogenesis. Our recent study with plasma EVs from HIV positive smokers has revealed some exciting information. First, we have shown the downregulation of properdin in the plasma EVs from HIV positive smokers, a protein in the human complementary system that interacts with viral proteins, suggesting a relatively high risk of secondary infections [49]. We also observed an alteration in the levels of CD9, CETP, and vitronectin (VTN) in plasma EVs from the HIV positive population; however, further studies are required to reach to any conclusion. We have also looked at the differential cytokine packaging within the same population [37]. We identified two cytokine/chemokines, IL-6 and MCP-1, which were significantly packaged within plasma EVs from HIV-infected smokers [37]. We further confirmed this finding by measuring their concentrations in the EVs derived from macrophages exposed to CSC and HIV, where IL-6 showed a similar trend (manuscript under review). These findings suggest that: a) EVs can potentially be used as a biomarker for smoking-mediated HIV pathogenesis and b) modulating EV-mediated HIV pathogenesis has potential for therapeutic intervention. Several other studies are also looking at potential biomarkers to indicate the possible association between HIV and tobacco smoking, as well as to predict the development of non-AIDS-related illnesses in HIV positive smokers [128,129,130]. Further, Il-6 stands out as a pro-inflammatory cytokine, which is elevated in HIV smokers [131]. In addition, Steel at el. have suggested that the chemokine RANTES could be one of the potential candidate biomarkers, which was elevated in their study with HIV positive smokers [128]. Our studies have also demonstrated that these agents are also highly packaged in EVs in the presence of HIV and smoking. Moreover, soluble CD14 and transforming growth factor beta (TGF-β1) are also reported to be elevated in virally suppressive HIV positive smokers [129]. Soluble CD14 is a biomarker for monocyte activation, which is also associated with cardiovascular disease [132]. On the other hand, TGF-β1 has a crucial role in regulating cells of immune system, in which increasing TGF-β1 was correlated to a defective T cell proliferation in an HIV-infected population [133]. Among these two, TGF-β1 is reported to be in EVs, however confirming its potential as a biomarker for smoking-mediated HIV progression warrants further investigation [134]. In recent years, more studies with regard to EVs in HAND have revealed that EVs influence the CNS, ultimately deteriorating HAND [21,120,135,136,137,138]. For example, HIV-induced microRNA-7 and cathepsin B were shown to be transported by EVs to neurons, resulting into neuronal damage [21,135]. Additionally, as previously mentioned, Pulliam et al. have identified several other biomarkers of neurodegeneration, which include L1CAM, Aβ, NF-L, and HMGB1, in the neuron-derived EVs from individuals with HAND [120]. Although cigarette smoking is postulated to be associated with exacerbated HIV and HAND pathogenesis, to our knowledge, to date no biomarker has been identified that could indicate the development of HAND in HIV positive smokers. A summary of the discussions above is summarized in Table 1.

## 5. Therapeutic Potential of Using EVs in HIV-Tobacco Smoking Comorbidity 

We have already demonstrated the role of EVs in carrying pro- and anti-oxidant contents, functional molecules relevant to smoking and exacerbation of HIV and HAND. Evidently, EVs are gaining importance as biological vehicles to carry various molecules, including drugs. EVs are a natural nanocarrier, low immunogenic in nature, with semi-selective targeting capacity. Thus, EVs are capable of evading phagocytosis or engulfment by macrophages and lysosomal degradation and vascular occlusion, thereby circulating within the body for a longer time [139,140]. Moreover, as EVs potentially evade first pass metabolism, they have a greater potential of reaching target organs [138]. Therefore, the therapeutic potential of EVs in smoking and HIV can be discussed from two perspectives: a) EVs as drug carriers and b) EVs in biomarker identification.

### 5.1. EVs as Drug Carriers

#### 5.1.1. In the Periphery 

EV drug loading capacity and efficiency has been explored, mostly in the case of anti-cancer drugs, to induce immunity against tuberculosis, toxoplasmosis, and diptheria toxoid, for delivery of small interfering RNA (siRNA) and much more [139,141,142,143,144,145,146]. Undoubtedly, EVs are a highly promising option as a drug delivery system. However, loading EVs with anti-retroviral drugs (ART) to target HIV is barely studied. Recently, Zou et al. have demonstrated the efficacy of engineered EVs loaded with curcumin (chemodietary agent that suppresses HIV replication) or miR-143 (apoptosis-inducing miRNA) to suppress HIV replication in vitro and ex vivo systems of latent HIV reservoirs, which also successfully suppressed HIV envelope protein-expressing solid tissue tumor in a humanized mouse model [147]. siRNA-based therapy is also being largely explored to target the latent HIV reservoirs. Although highly promising, siRNA are short-lived and not well-equipped to penetrate major reservoirs [148]. Packaging siRNA in EVs can overcome these issues. In fact, mesenchymal stem cell and dendritic cell-derived EVs have already been utilized to deliver siRNA for other purposes [149,150]. Again, based on earlier studies, EVs can package CYP enzymes, which could play a major role in drug metabolism and oxidative stress [97,151]. Therefore, targeting heightened CYP levels in EVs by selective CYP inhibitors could be a way to neutralize the increased drug metabolism/drug resistance and subsequent oxidative stress. In a similar way, EVs loaded with AOEs can reduce smoking-exacerbated HIV replication mediated by oxidative stress. EVs could also be used in formulating personalized therapy by isolating EVs from the patient, which will significantly reduce the risk of immunogenicity. 

#### 5.1.2. In CNS

The ability of EVs to cross the BBB makes them efficient vehicles to reduce the burden of HAND. EVs, especially derived from macrophages, can readily cross BBB and deliver their cargos to resident macrophages [30]. EVs can be loaded with cART drugs to improve their efficacy in smoking-exacerbated, HIV-associated comorbidities, including HAND. Again, long-term usage of cART drugs can lead to CNS toxicity, in part via oxidative stress pathway [152,153]. Loading of EVs with the AOE, catalase, has already shown neuroprotective effects in *in vitro* and *in vivo* models of Parkinson’s disease [154]. Similarly, antioxidants in combination with ART can be loaded in EVs, which could be used as potential interventions in smoking-exacerbated HAND. Additionally, dendritic cell-derived EVs have been modified to deliver siRNA therapy to cross the BBB, which could provide a promising means of targeting HIV reservoirs in the brain [150]. 

#### 5.1.3. Targeting EVs

EVs possess naturally semi-selective targeting properties, depending on their origin and composition. Although not completely determined yet, adhesion molecules like integrins, major histocompatibility complex class II molecules, tetraspanin complexes, lipid compositions, glycans, and liposomes found on the surface or inside the vesicles, are demonstrated to aid in targeting [91,155,156,157]. Several studies have suggested that EVs attain the homing pattern of their originating cells, suggesting that it is crucial to consider this natural tropism of EVs while developing therapeutic carriers [158,159]. Initiatives have been taken to develop engineered EVs to more specifically bind to the target cells by enriching with specific antibodies, nanobodies, peptides, nucleic acids, as well as using the pseudotyping method commonly used in altering viral tropism [145,160], further discussed in the review by Murphy et al. [91]. However, not many studies were performed to target EVs to combat HIV viral infection in the periphery and in CNS cells, let alone under the influence of cigarette smoking. Recently, the study performed by Zou et al. designed an HIV specific monoclonal antibody containing EVs, loaded them with curcumin/miRNA, which successfully targeted HIV reservoirs (in vitro and ex vivo) as well as an in vivo study to reduce viral replication [147]. Similarly, modifying EVs to target cellular reservoirs e.g. CCR5, CXCR4 co-receptors, and CD4, could be the next step to deliver targeted cART in these cells. As eradication of viral reservoirs remains a big challenge in HIV, EVs could potentially play a significant role in specifically targeting these populations.

### 5.2. EVs in Biomarker Identification

#### 5.2.1. EVs in HIV

EV contents could serve as biomarkers for HIV, either for diagnosis or prognosis. Recognition of specific protein and RNA contents in plasma EVs from HIV positive population has been used for diagnostic and prognostic purposes, as well as for the determination of treatment efficacy [161]. Identifying specific miRNA levels in EVs has shown significant promise in this regard. EVs isolated from a small group of HIV-suppressed patients demonstrated that miR-21 was downregulated in HIV controlled patients, with a decreasing pattern of CD4 cell count [162]. In addition, EVs obtained from an HIV positive population without antiretroviral therapy are larger in size and greater in quantity, with a lower amount of miRNA-155 and miRNA-223 [111,163]. These findings suggest that EVs could potentially be used to indicate the stages of infection, as well as markers of response towards treatment. As mentioned earlier, neuron-derived EVs, isolated from the plasma, could serve as an excellent source of liquid biopsy biomarkers for identifying neurocognitive disorders in HIV [121,164]. Additionally, urinary EVs collected from HIV positive individuals may provide a good source of HIV biomarkers, including p24 [165]. In fact, commercial diagnostic tools are also being developed to identify HIV in patients [161,166]. Moreover, PLWHA have a significantly higher rate of neurocognitive disorders. As mentioned, Aβ deposition is an indicator of Alzheimer’s disease, which was also identified in the EVs of HIV positive individuals, suggesting the possibility of using Aβ in EVs as a biomarker [119]. 

#### 5.2.2. EVs in HIV and Smoking Comorbidity

EVs have significant potential to be used as biomarkers of smoking-induced HIV-progression in a non-invasive way. EVs obtained from smokers could potentially be used in the diagnosis of smoking-induced diseases. Ryu et al. have summarized the potential biomarkers for lung cancer, chronic obstructive pulmonary disease, cardiovascular disease, and Non-small cell lung cancer, in the presence of cigarette smoke, which could be indicated by identifying miRNAs and protein content present in EVs derived from plasma, serum, bronchoalveolar lavage (BAL), and urine collected from patients [167]. 

In addition, a few possible markers, IL-6, RANTES, soluble CD14, and properdin have been identified within EVs isolated from the plasma of HIV smokers [37,49,128,129,131,132,133,134]. However, further studies are warranted to establish their true potential as biomarkers of smoking-induced HIV pathogenesis. Moreover, future investigations to discover biomarkers in plasma EVs, which are altered only in the presence of both HIV and cigarette smoking, will also be highly beneficial. In addition, studies to link cigarette smoking and HIV with HAND progression, as well as biomarker discovery, will strengthen our understanding of the gravity of the impact that smoking has on HIV. 

**Figure 2:**
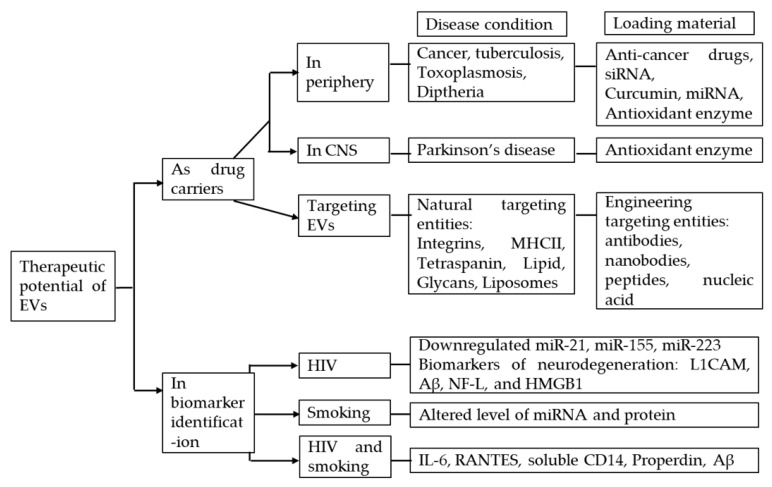
Therapeutic potential of using EVs in HIV-tobacco smoking comorbidity. siRNA: Small interfering RNA; miRNA: microRNA; MHCII: major histocompatibility complex II; L1CAM: L1 cell adhesion molecule; Aβ:Amyloid beta; Neurofilament Light; HMGB1: High mobility group box 1; IL-6: Interleukin 6; RANTES: Regulated on activation, normal T cell expressed and secreted; CD14: Cluster of differentiation14

Taken together, understanding the role of EVs in smoking-exacerbated conditions in HIV patients may provide opportunities to develop new therapeutic interventions for this subgroup of the HIV population. Figure 2 briefly summarizes the therapeutic potential of EVs.

## 6. Conclusion and Future Prospects

To address the questions initially proposed in this review, EVs are undoubtedly a major player in smoking-mediated HIV and HAND progression. It is yet to be determined how and to what extent EVs carry oxidative stress and inflammatory agents across the BBB and within the CNS as well as cause HIV pathogenesis and neuronal damage, especially in the presence of tobacco smoking. The prospect of EVs as biomarkers and therapeutic carriers is highly promising. However, in terms of the knowledge and potential of EVs in influencing smoking-mediated HIV pathogenesis and HAND, investigations are still at the very early stages. Therefore, there is a tremendous scope of research in this field. 

EVs, particularly from macrophages, have the advantage of penetrating the highly selective, semipermeable BBB, allowing them a unique exposure to the resident cells of the CNS [30]. EVs from uninfected and HIV-infected macrophages, in the absence and/or presence of tobacco exposure, can potentially deliver oxidative (e.g. CYPs) and inflammatory elements (cytokines/chemokines) to the brain and exacerbate HIV pathogenesis and HAND. Therefore, future studies to investigate the role of EVs in CNS cells would be desirable. Further investigations to identify the specific role of EVs containing cytokines and chemokines (especially IL-6), as well as CYPs, AOEs, other proteins, mRNA, and miRNA on HIV and HIV-associated neuropathogenesis, are highly desirable. 

Researchers have demonstrated the potential of EV-carrying AOEs to negate the mediation of oxidative stress in a CNS disease. However, in the case of inhibiting viral transfection by EVs-carrying agent, more studies are warranted. Thus, the use of EVs as therapeutic carriers in HIV viral suppression or targeting latency reservoirs is highly promising. 

Similarly, EVs have a high potential in HIV infection stage diagnosis, prognosis, as well as biomarker discovery for smoking-mediated HIV and HAND progression. The potential of EVs is already under investigation in terms of biomarker identification, diagnosis, and prognosis of various conditions, and as therapeutic carriers of drugs in the periphery as well as in the CNS. Our studies and others have postulated the prospect of IL-6, RANTES, soluble CD14, and properdin being potential biomarkers for tobacco-enhanced HIV-pathogenesis [37,49,128,129,131,132,133,134]. Whether they could be used in actual therapeutic settings will require further study. 

Therefore, knowledge about EVs can help us in devising novel therapeutic agents for treating tobacco-induced HIV and HIV-associated pathologies more efficiently. Future studies should be directed towards finding the mechanistic pathways to fully comprehend the role of EVs in smoking-mediated HIV and HAND pathogenesis, which can be employed to develop the clinical and therapeutic potentials of EVs. 

## Figures and Tables

**Table 1:** Role of extracellular vesicles in HIV, tobacco smoking, and HIV and smoking.

HIV-Associated EV Component		HIV + Smoking-Associated Component	
Type	Specific	Type	Specific
Viral proteins	Nef, Tat [104]	Cytokines	IL-6 ^10^, MCP-1 ^11^, RANTES ^12^ [37,128,131]
Viral entry receptors	CCR5 ^1^ [103]	Others	Properdin [49], soluble CD14^4^, TGF-β1 ^13^ [129]
Oxidative stress markers	cystine, oxidized cys-gly, Catalase, PRDX1 ^2^, PRDX2 ^2^, and TXN ^3^ [115]		
Anti-inflammatory marker	PUFA [115]		
Immune activation markers	CD14 ^4^, CRP ^5^, HLA-A, and HLA-B ^6^ [115]		
NeurodegenerationMarkers	Aβ ^7^, HMGB1 ^8^ and NF-L ^9^ [119,120,121]		

^1^ C-C chemokine receptor type 5; ^2^ Peroxiredoxin 1 and 2; ^3^ Thioredoxin-1; ^4^ Cluster of differentiation14; ^5^ C-reactive protein; ^6^ human leukocyte antigen major histocompatibility complex, class I, A & B; ^7^ Amyloid beta; ^8^ High mobility group box 1 protein; ^9^ Neurofilament light; ^10^ Interleukin 6; ^11^ Monocyte chemoattractant protein-1; ^12^ Regulated on activation, normal T cell expressed and secreted; ^13^ Transforming growth factor beta-1.
